# Clinical impact of CD200 expression in patients with acute myeloid leukemia and correlation with other molecular prognostic factors

**DOI:** 10.18632/oncotarget.4901

**Published:** 2015-08-18

**Authors:** Daniela Damiani, Mario Tiribelli, Donatella Raspadori, Santina Sirianni, Alessia Meneghel, Margherita Cavalllin, Angela Michelutti, Eleonora Toffoletti, Antonella Geromin, Erica Simeone, Monica Bocchia, Renato Fanin

**Affiliations:** ^1^ Division of Hematology and Bone Marrow Transplantation, Azienda Ospedaliero-Universitaria di Udine, Udine, Italy; ^2^ Division of Hematology, University of Siena, Siena, Italy

**Keywords:** CD200, acute myeloid leukemia, prognosis, survival

## Abstract

CD200, a protein belonging to the immunoglobulin superfamily, has been associated with a poor prognosis in lymphoproliferative disorders and in acute leukemia. We studied the expression of CD200 in a series of 244 patients with diagnosis of acute myeloid leukemia (AML), to evaluate its impact on outcome and its possible association with other known prognostic factors.

CD200 was found in 136/244 (56%) patients, in 41 of whom (30%) with high intensity of expression (MFI ≥ 11). CD200 was more frequent in secondary compared to de novo leukemia (*p* = 0.0006), in CD34 positive cases (*p* = 0.00001), in Bcl2 overexpressing cases (*p* = 0.01), in those wild-type Flt3 (*p* = 0.004) and with favorable or unfavorable compared to intermediate karyotype (*p* = 0.0003). CD200+ patients have a two-fold lower probability to attain complete remission, both in univariate (*p* = 0.006) and multivariate (*p* = 0.04) analysis. The negative impact of CD200 was found also in overall survival (*p* = 0.02) and was correlated with the intensity of expression of the molecule (*p* = 0.024). CD200 has an additive negative impact on survival in patients with unfavorable cytogenetic (*p* = 0.046) and in secondary leukemia (*p* = 0.05), and is associate with a worsening of outcome in patients with favorable biological markers, such as mutated NPM (*p* = 0.02), wild-type Flt3 (*p* = 0.034), negativity of CD34 (*p* = 0.03) and of CD56 (*p* = 0.03).

In conclusion, CD200 is emerging as both a prognostic factor and a potential target of novel therapeutic approaches for AML, aiming to reverse the “do not eat me” signal of CD200 or to manipulate the suppressive immune microenvironment induced by CD200 binding to its receptor.

## INTRODUCTION

Despite relatively high rates of complete remission (CR) with induction and consolidation chemotherapy, relapse occurs in the majority of adult patients with acute myeloid leukemia (AML), accounting for the poor likelihood of long-term survival. [1, 2]

Besides clinical and laboratory features at diagnosis, such as age, performance status, tumor burden, antecedent hematological disorder or prior exposure to chemotherapy and extramedullary disease [3–6], various cytogenetic or molecular abnormalities are currently used for risk stratification [7, 8]. The genetic features of AML, identifies in the last decades along with the understanding of leukemia biology, are pivotal to move towards a patient-tailored therapy and follow-up. [9–13] However, to date only little improvement in survival rate has been observed, especially in high-risk patients, despite a wider use of stem cell transplantation (SCT), that is still considered the best consolidation therapy in poor-risk AML for the graft-versus-leukemia action of allogeneic T- cells [14–19].

Given these premises, it is clear that mechanisms by which tumor cells can hamper immune recognition and survive in a permissive microenvironment should be taken into account. CD200 is a type-1 membrane glycoprotein containing two immunoglobulin domains, normally expressed in a broad range of cells. [20] Its binding with CD200R is able to induce an immunosuppressive signal and, in animal models, favors the tumor growth [21–25]. In hematological malignancies CD200 expression was first reported in chronic lymphocytic leukemia, where it has a role in differential diagnosis with mantle cell lymphoma [26]. Lack of CD200 expression in plasma cells has been associated with more aggressive multiple myeloma. [27] More recently CD200 aberrant expression has been proposed as an adverse prognostic factor in AML. [28]

Since the relative paucity of data and the somehow conflicting results, aim of our study is to assess the pattern of CD200 expression in a series of adult patients with AML, its association with other known prognostic factors and the possible impact on clinical outcomes.

## PATIENTS AND METHODS

Two hundred forty-four patients with diagnosis of non-promyelocytic AML admitted at the Divisions of Hematology of Udine and Siena between January 2008 and June 2014 were included in the study. Clinical and biological characteristics at diagnosis are summarized in Table 1. Diagnosis was performed on bone marrow smears according to FAB classification. [29] Cytogenetic risk was classified according to MRC criteria [30]. Flt3 and NPM mutations were evaluated as previously described. [31, 32] Combined molecular and cytogenetic risk was assigned according to Döhner. [9]

**Table 1:** Clinical/biological characteristics at diagnosis

	*n*. 244
**Età Median (range) yrs** **Età ≥ 55**	59 (18–84) 147
**Sex: M/F**	173/71
**WBCx10^9^/L: mean ± 2SD** **WBC ≥ 30 × 10^9^/L**	13.9 ± 42.5 98 (40%)
**Type of leukemia : De novo** **Secondary**	173 (71%) 71 (29%)
**FAB (de novo): M0** **M1** **M2** **M4** **M5** **M6**	10 (6%) 32 (18%) 28 (16%) 35 (20%) 65 (38%) 3 (2%)
**Combined cytogenetic/molecular risk**: **Favorable** **Int-1** **Int-2** **Unfavorable** **NA**	43 (18%) 61 (25%) 35 (14%) 70 (29%) 35 (14%)
**Flt3-ITD: mutated** **wild type** **NA**	46 (19%) 170 (70%) 28 (11%)
**NPM: mutated** **wild type** **NA**	65 (27%) 145 (59%) 34 (14%)
**Cytogenetic risk**: **Favorable** **Intermediate** **Unfavorable** **NA**	14 (6%) 136 (56%) 70 (29%) 24 (10%)
**CD34: positive** **negative**	130 (53%) 114 /47%)
**CD56: positive** **Negative** **NA**	87 (36%) 154 (63%) 3 (1%)
**Bcl2-MFI: ≥ 17** **<17** **NA**	124 (51%) 113 (46%) 7 (3%)

Blast cells immunophenotype was evaluated by multiparametric flow cytometry (Facs Diva II, BD). CD200 aberrant presence on blast cells was tested using PE anti-human CD200- antibody (BD Pharmingen, Brussels, Belgium) and expressed as the percentage of positive cells (with 20% as cut-off value) and as the mean fluorescence intensity (MFI) obtained by the ratio of fluorescence intensity of the test and of its isotypic control. Cases with a MFI = 1 were considered negative, patients with a MFI < 11 as “low expressing” and case with MFI ≥ 11 were considered as “high expressing”.

Patients were treated according to Ethic Board approved Institutional protocols with induction chemotherapy and at least two consolidation courses after complete remission (CR). High-risk cases (defined as at least one of the following: secondary AML, poor response to induction chemotherapy, unfavorable cytogenetic or combined genetic risk, or patients with early relapse) underwent allogeneic stem cell transplantation (SCT) from related or unrelated donor. Poor performance status or elderly patients, deemed not suitable for intensive chemotherapy, received cytoreduction with hydroxyurea, low-dose cytarabine or oral 6-mercaptopurine.

### Definitions and statistical analysis

Complete remission (CR) was defined as the complete peripheral hematological recovery and the absence of bone marrow disease (at morphological, immunophenotypic or molecular evaluation). Overall survival (OS) was calculated from diagnosis to death (irrespective from the cause). Leukemia free survival (LFS) was defined as the time between CR and relapse. Patients lost to follow up were censored at the time last seen alive.

Categorical variable were compared with Fisher exact test or Yates corrected chi square test, as required. Comparisons between continuous variables were evaluated by T student test or by Kruskall Wallis test. Factors affecting CR were assessed by univariate and multivariate logistic regression, and expressed as HR (95%CI). OS curves were constructed by Kaplan Meier method and differences among groups calculated by log-rank test. The Cox proportional hazard regression model was used to examine the potential prognostic factors for OS: all variables with *p* values ≤ 0.10 in univariate analysis were included in the multivariable model and a backward stepwise procedure was applied to identify significant factors. All *p*-values are 2-sided at a significance level of 0.05. Statistics was performed by NCSS 10 Statistical Software (2015) (“NCSS, LLC. Kaysville, Utah, USA, http://ncss.com/software/ncss.”).

## RESULTS

Aberrant expression of CD200 was found in 136/244 patients (56%) with a mean MFI of 11 (range 2–100). High intensity of expression (mean MFI = 23.5 ± 10) was detected in 41/136 (30%) positive patients. As shown in Table 2, CD200 was more frequently expressed in secondary leukemia (52/71, 73%), compared to de novo (84/172, 49%; *p* = 0.0006), while no association was found with age, WBC count at diagnosis and FAB subtype. However CD200 was more frequently expressed in CD34 positive blast cells (*p* < 0.00001) and in patients with high levels of Bcl2 (*p* = 0.01), while there was an inverse correlation with CD56 expression (39/87, 45% in CD56+ vs 95/154, 62% in CD56 negative patients; *p* = 0.015).

**Table 2:** CD200 and clinical/biological characteristics at diagnosis

	CD200+	*P*
**Age: ≥ 55 yrs** **< 55 yrs**	84/147 (57%) 53/97 (55%)	0.79
**Type of leukemia : De novo** **Secondary**	84/172 (49%) 52/71 (73%)	0.0006
**Cytotype: M0** **M1-M2** **M4-M5** **M6**	7/16 (43%) 35/65 (54%) 56/111 (50%) 1/3 (33%)	0.8
**WBC: ≥ 30 × 10^9^/L** **< 30 × 10^9^/L**	48/99 (48%) 87/145 (60%)	0.08
**Cytogenetic: Favorable** **Intermediate** **Unfavorable**	13/14 (93%) 65/139 (47%) 44/67 (66%)	0.0003
**Flt3-ITD: Positive** **Negative**	17/46 (37%) 105/170 (62%)	0.004
**NPM: Wild type** **Mutate**	99/145 (68%) 19/65 (29%)	0.0013
**Cytogenetic/molecular risk: Favorable** **Intermediate-1** **Intermediate-2** **Unfavorable**	23/44 (52%) 31/70 (44%) 20/36 (55%) 48/69 (69%)	0.02
**CD34: positive** **Negative**	99/129 (77%) 36/113 (32%)	<0.00001
**CD56: positive** **negative**	39/87 (45%) 95/154 (62%)	0.015
**Bcl2 MFI: ≥ 17** **<17**	65/101 (64%) 66/136 (48%)	0.01

High frequency of CD200 expression was detectable in patients without Flt3-ITD mutation (105/170, 62%) compared to patients with Flt3-ITD mutation (17/46, 37%, *p* = 0.004) and in patients with wild type NPM (99/145, 68%) vs those with mutated NPM (19/65, 29%, *p* = 0.0013).

Considering karyotype, a lower frequency of CD200 positivity was found in intermediate cytogenetic group (65/139, 47%), compared to favorable (13/14, 93%) and unfavorable risk group (44/67, 66%; *p* = 0.0003), and in the favorable or intermediate groups of the combined cytogenetic/molecular classification (74/150, 49%) compared to the unfavorable (48/69, 69%; *p* = 0.02).

### CD200 and response to induction therapy

One hundred forty-nine out of 244 (61%), obtained CR, 10/244 (4%) died during induction and 85/244 (35%) were resistant to induction therapy. Relapse occurred in 54/149 (36%) patients at a median of 30 months.

Factors affecting CR probability are listed in Table 3. In univariate analysis, age higher than 55 years (*p* < 0.00001), secondary disease (*p* < 0.00001), CD34 positivity (*p* = 0.0001) and unfavorable cytogenetics (*p* = 0.01) or unfavorable molecular/cytogenetic status (*p* = 0.00001) were associated with reduced probability to achieve CR. CR was obtained in 73/130 (56%) in CD200+ and in 76/100 CD200- (76%) evaluable patients (*p* = 0.006). Patients with aberrant CD200 expression have almost two fold less probability to obtain CR (OR = 0.45, 95% CI: 0.26–0.80).

**Table 3:** uni and multivariate analysis of potential factors for CR

	Univariate		Multivariate	
	OR (95%CI)	*p*-value	OR (95%CI)	*P*-value
**Age ≥ 55 yrs**	0.23 (0.12–0.43)	< 0.00001	0.40(0.19–0.87)	0.0019
**WBC ≥ 30 × 10^9^**	0.6 (0.36–1-11)	0.1		
**Secondary leukemia**	0.19 (0.10–0.35)	< 0.00001	0.31(0.15–0.65)	0.0019
**Unfavorable Cytogenetic**	0.08 (0.005–0.61)	0.01		
**Unfavorable Cytogenetic/molecular**	0.07 (0.025–0.24)	0.00001	0.12(0.03–0.47)	0.0025
**NPM unmutated**	0.26 (0.12–0.55)	0.0004		
**CD34 positive**	0.27 (0.15–0.48)	0.0001	0.36(0.17–0.77)	0.008
**CD56 positive**	0.74 (0.42–1.30)	0.27		
**CD200 positive**	0.52(0.29–0.91)	0.02		
**CD200 MFI ≥ 11**	0.41 (0.19–0.92)	0.03	0,49(0.20–0.95)	0.040
**Bcl-2 MFI ≥ 17**	0.78 (0.45–1.35)	0.38		

The negative impact of CD200 on remission probability was maintained in multivariate analysis (*p* = 0.04), along with more conventional factors such as age (*p* = 0.002), type of leukemia (*p* = 0.002), CD34 positivity (*p* = 0.008) and unfavorable cytogenetic risk (*p* = 0.0025) (Table 3).

### Overall survival

**Figure 1:**
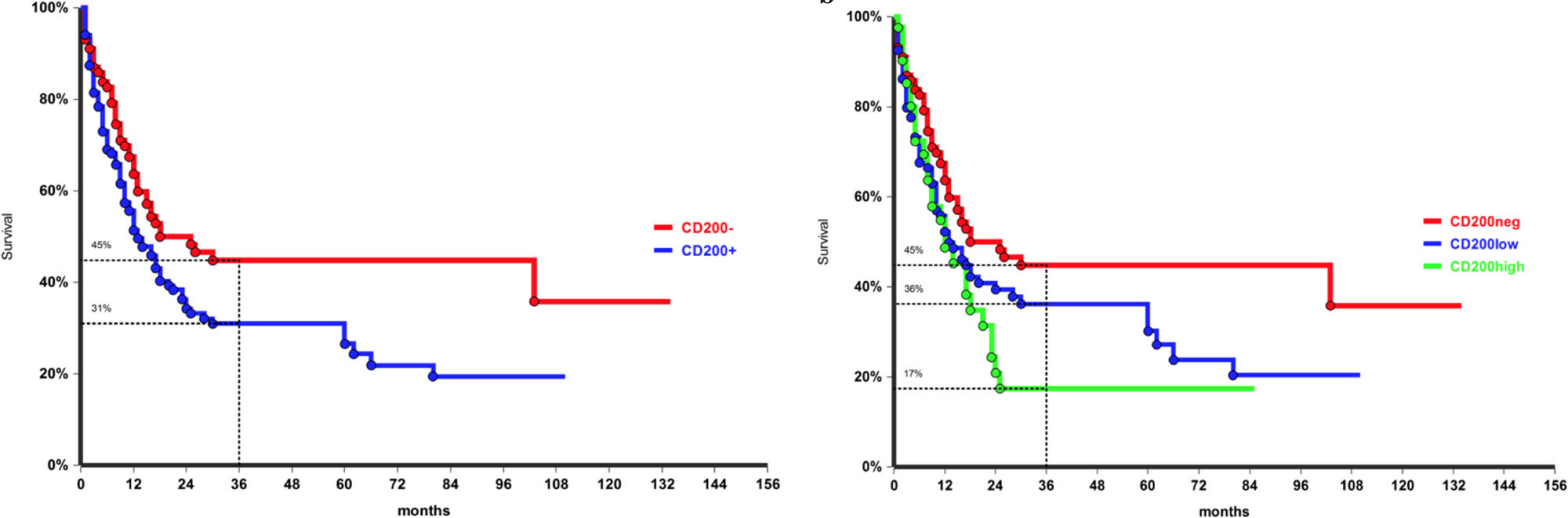
Overall survival of the entire population according to CD200 expression, *p* = 0.02, **a.** and by CD200 intensity of expression, *p* = 0.024, **b.** CD200 negative: MFI = 1; CD200 low: MFI < 11; CD200 high: MFI ≥ 11

At the time of analysis 101/244 patients (41%) were alive without evidence of disease, with a 3-year survival probability of 37% (95%CI: 29–43). Three-year OS was significantly reduced in patients aged ≥ 55 years compared to younger patients (21% vs 60%, *p* < 0.0001), in case of secondary AML (20% vs 43% in de novo leukemia, *p* = 0.0004), and in CD34 positive cases (23%, vs 53% in CD34- patients, *p* < 0.0001). As expected, unfavorable karyotype was associated with poorer survival (3-year OS 22%, compared to 40% in normal/intermediate and 60% in favorable cytogenetic, *p* = 0.0003). No impact on OS was also observed for Flt3-ITD, both in the whole population and in the normal/intermediate cytogenetic subgroup. Conversely the presence of a NPM mutation conferred a survival advantage irrespective to karyotype with a 3-year OS 50% in mutated vs 33% in WT (*p* = 0.01). Considering the combined cytogenetic/molecular risk, OS in the unfavorable group (30% at 3 years) was significantly lower compared with the other risk groups (58% in favorable, 43% in intermediate-1, 34% intermediate-2; *p* = 0.0001). With regard of CD200, OS was negatively affected by both aberrant molecule expression (3-year OS 31% vs 45%; *p* = 0.02, Figure 1a), and by intensity of expression (17% in patients with high MFI compared to 36% in those with low intensity of expression; *p* = 0.024) (Figure 1b).

In multivariate analysis (Table 4), advanced age (HR = 0.46, 95%CI 0.30–0.69), unfavorable cytogenetic (HR = 0.33, 95%CI 0.13–0.79), CD34 positivity (HR 0.44, 95%CI 0.29–0.67) and CD200 MFI ≥ 11 (HR 0.57, 95%CI 0.32–0.97) retained their negative prognostic role.

**Table 4:** multivariate analysis of factors for OS

	HR (95%CI)	*P*
**Age ≥ 55 yrs**	0.46 (0.30–0.69)	0.0002
**Secondary leukemia**	0.92 (0.61–1.39)	0.78
**Unfavorable cytogenetic risk**	0.33(0.13–0.79)	0.01
**CD34 positivity**	0.44 (0.29–6.67)	0.0002
**CD200 MFI ≥ 11**	0.59 (0.32–0.97)	0.04

### Association of CD200 with other prognostic factors and outcome

We then evaluated the impact of the expression of CD200 in different biologically/molecularly defined prognostic groups.

**Figure 2:**
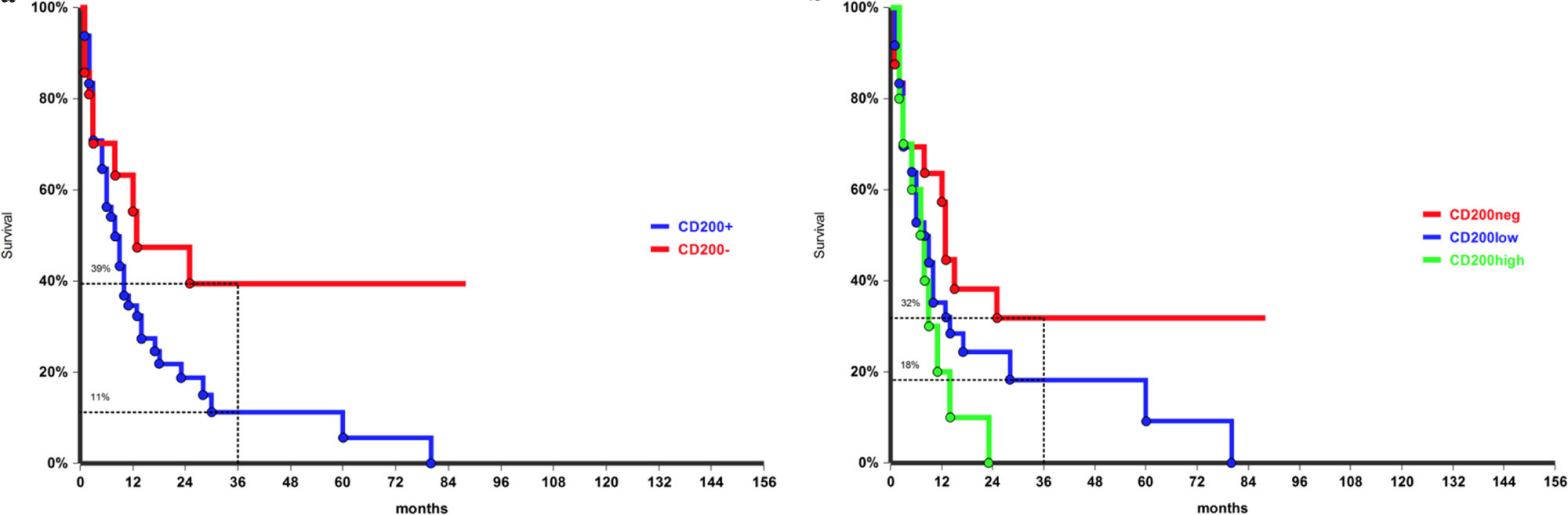
Overall survival in patients with unfavorable cytogenetics: difference in CD200 negative and in CD200 positive patients, *p* = 0.046, **a.** and according to CD200 MFI, *p* = 0.06, **b.**

Patients with unfavorable karyotype expressing CD200 have a survival rate significantly lower than CD200 negative patients (3-year OS 11% vs 39%, *p* = 0.046) (Figure 2a). We found a trend for a lower OS according to CD200 intensity of expression: 3-year survival rate was 18% in CD200-low compared to 0% in CD200-high expressing patients (Figure 2b). A worse survival was observed also in patients with favorable cytogenetic and high CD200 expression (33%) compared to those CD200-low (79%). The lack of statistical significance is probably due to the small size of the two groups. Instead, CD200 positivity did not influence survival probability in the intermediate cytogenetic risk.

**Figure 3:**
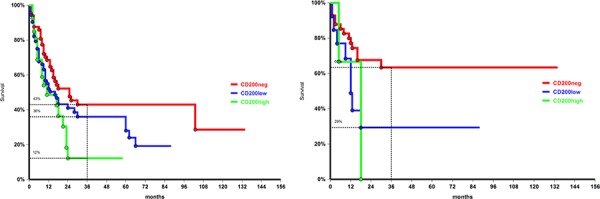
Overall survival by CD200 intensity of expression in patients FLT3-ITD negative *p* = 0.034, **a.** and in NPM mutated group, *p* = 0.02, **b.**

Considering Flt3, co-expression of CD200 did not worsen survival probability in Flt3-ITD+ patients, but negatively affected survival in ITD-negative patients; 3-year OS was 42% in CD200-, 36% in CD200-low and 0% in CD200-high patients (*p* = 0.034, Figure 3a). In NPM mutated patients CD200 was less frequent than in NPM WT ones, but, when present, CD200+/NPM mutated cases had a lower OS rate compared to their CD200- counterpart (3-year OS 63% vs 25%, *p* = 0.02, Figure 3b). In NPM-WT patients 3-year OS was 29% in CD200+ and 37% in CD200- cases (*p* = 0.1).

**Figure 4:**
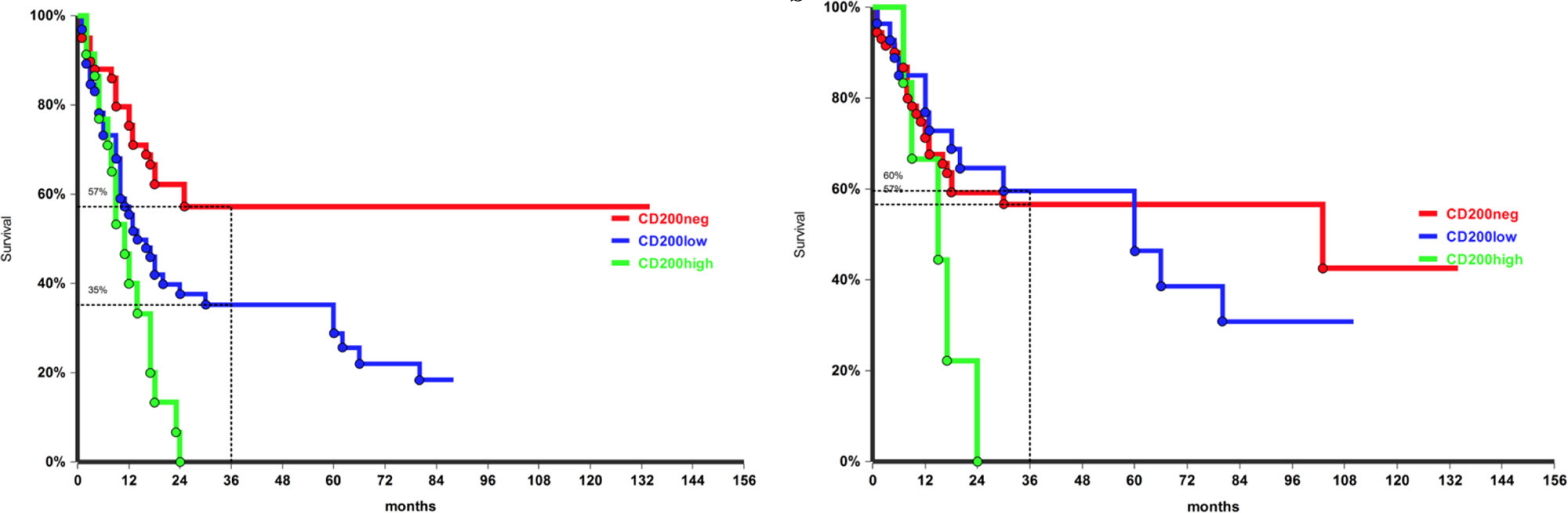
Overall survival according to CD200 MFI in CD56 negative patients, *p* = 0.04, **a.** and in CD34 negative patients, *p* = 0.03, **b.**

No difference in survival probability was found according to CD200 status in CD56+ patients. Conversely in CD56- AML, CD200 expression was associated with a lower survival probability: 3-year OS was 57% in CD200-, 35% in CD200-low and 0% in CD200-high patients (*p* = 0.03; Figure 4a). As for CD56, no impact on survival was observed for CD200 in CD34+ patients, while in the CD34-negative group CD200 high was associated with worse 3-year OS probability: 0% compared with 59% in CD200 low and 57% in CD200 negative patients (*p* = 0.03, Figure 4b).

**Figure 5:**
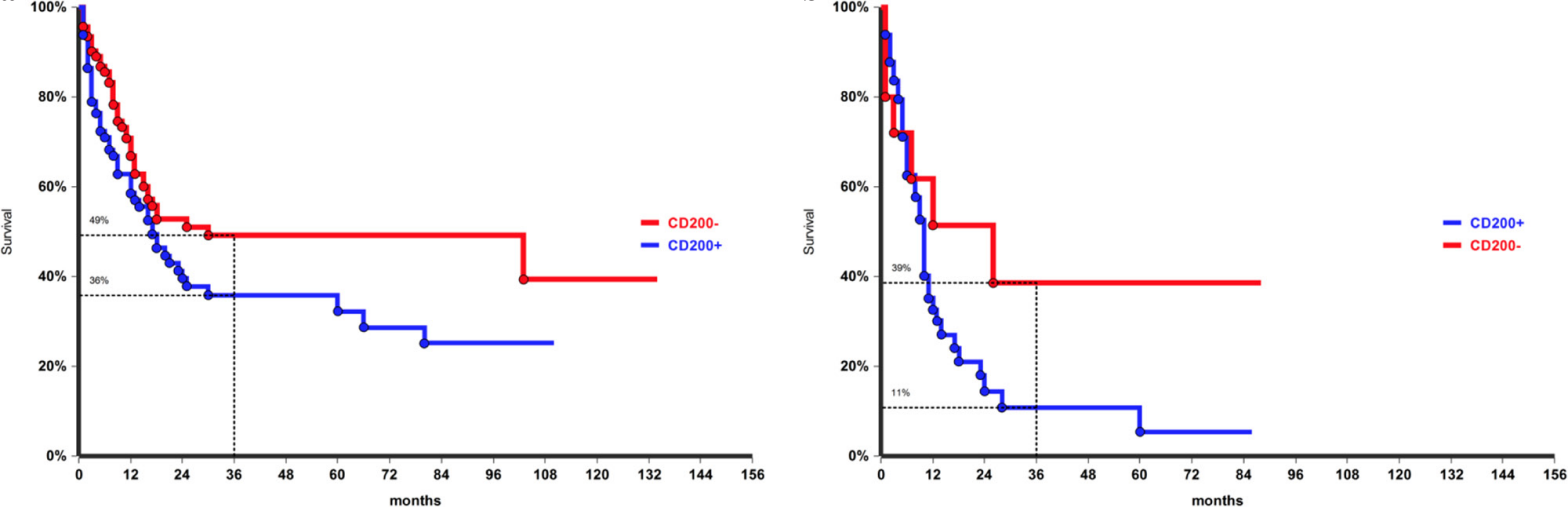
Overall survival by CD200 expression in de novo, *p* = 0.04 **a.** and secondary leukemia, *p* = 0.05 **b.**

Last, CD200 had a negative impact on OS in both de novo and in secondary AML. At 3-year, in de novo cohort OS was 49% in CD200- and 35% in CD200+ patients (*p* = 0.04; Figure 5a). In secondary AML 3-year OS was 38% in CD200- and 16% in CD200+ (*p* = 0.05; Figure 5b).

## DISCUSSION

Along with factors promoting drug resistance or favoring neoplastic cells’ escape from programmed death, the mechanisms by which AML can evade immunosurveillance have recently gained new attention. Many ways are employed by leukemic cells to escape recognition and destruction by immune effector cells, such as down-regulation of HLA molecules, secretion of inhibitory cytokines, recruitment of tolerogenic cells, modification of costimulatory and co-inhibitory signals. In the present paper we focused on the potential impact of CD200 expression on outcome of a series of 244 adult patients with non-promyelocytic AML. We found an aberrant expression of CD200 in 56% of cases, with a high intensity of expression in 30%. Differently from what has been reported by Tonks at al [28] we did not find differences in CD200 expression among FAB subtypes, but in our series 77% of CD34-positive expressed also CD200. Many authors reported an association between CD200 and stem cell properties in solid tumors [33–35], suggesting that cancer stem cells use CD200 system to prevent the attack of the immune system. In our cases we did not find association with other marker of leukemic stemness. As in the work by Tonks et al. [28], aberrant CD200 expression was detected in the vast majority (93%) of AML cases with favorable cytogenetic, but we found high rate of CD200 positivity also in patients with unfavorable katyotype (*P* = 0.0003), NPM1 wild type (*P* = 0.001), Flt3-ITD negative (*P* = 0.004), and with unfavorable cytogenetic/molecular status, as recently defined by Döhner [9] (*P* = 0.02). Moreover CD200 was more frequently expressed secondary than in de novo leukemia (73% vs 49%).

CD200 positivity was associated with lower rates of complete remission and survival, as confirmed in multivariable analysis. Tonks and coworkers [28] found a negative impact on survival in the whole population and the subgroup with core binding factor AML. Analyzing survival in the different cytogenetic risk groups, we found a negative impact of CD200 expression in patients with unfavorable karyotype, while in cases with CBF alterations the lack of significance is probably due to the limited number of patients. Interestingly, CD200 seemed to worsen survival in NPM mutated and in Flt3-ITD wild type patients, irrespectively of karyotype, so identifying a subset of patients with poor prognosis in a group usually associated with a more favorable outcome. A negative impact on OS was observed also in CD56 negative patients, that are usually considered to have a better prognosis compared to CD56+ patient. [36–38] Conversely, no differences in OS probability are evident evaluating CD200 and CD34 expression. It could be speculated that CD200 in CD34+ leukemic cell resembles the normal expression on CD34+ progenitors, where it contributes in protection from auto-aggression by the immune system cells. Despite the different rate of expression in de novo and secondary AML, CD200 expression had a negative impact on OS in both groups; 3-year survival in CD200+ de novo cases (36%) was similar to that of CD200- secondary AML (39%), and significantly lower than CD200- de novo (49%) but higher than CD200+ secondary (11%) patients, respectively. So, the combination of CD200 expression and leukemia type define three group of patients with significant different survival expectation (*p* = 0,0007).

Mechanisms by which CD200 exerts its negative influence on outcome are only partially defined. In the recent years the complex network connecting innate and adaptive immune cells to response to foreign pathogens, to self-antigens and to tumor cells begin to clarify, and their identification may contribute to the design of novel target therapies. Coles et al. observed a suppression of memory T-cells and a reduction of NK activity in CD200 positive AML patients, especially in the NK cells with high lytic activity [39, 40]. We did not find a significant reduction in NK populations in our population, but T-cells were studied on in a minority of cases, thus we cannot confirm the observation of an increased frequency of CD4+ regulatory cell and a recovery of Th1 response by Tregs depletion in CD200-positive leukemia [41]. In line with this findings, Memarian et al. reported high levels of IL10 production in autologous mixed lymphocyte reactions in presence of AML-dendritic cells [42]. Coles and coworkers have hypothesized a co-operation between CD200/CD200R and PD-L1/PD-1 axis as a cause of the worse prognosis linked to overexpression of CD200 and PD-L1 on leukemic blast cells [43].

In our series, preliminary data indicate a higher occurrence of myeloid-derived suppressor cells (MDSC), that are known to play a central role in regulating immune response and tumor tolerance [21, 44–46], in CD200 positive patients. Similar findings have been reported by Moertel et al. in human brain tumors [47]. They found that high levels of CD200 correlated with MDSC expansion and demonstrated, in an experimental model, that the block of CD200/CD200R prevents MDSC induction and inactivates the release of inhibitory cytokines. If confirmed, our data may be of interest not only for prognosis but also for the pathogenesis of AML. Chen et al have reported a role of MDSC in inducing multilineage cytopenia and cytological dysplasia [48].

In conclusion, our study confirms the negative prognostic role of CD200 in AML and identifies subgroups of patients in which CD200 significantly reduces survival probability. Of note, beside an additive negative impact in patients with unfavorable cytogenetic or secondary AML, CD200 overexpression is associated with a worse prognosis also in patients with biological markers considered favorable, such as mutated NPM, Flt3 wild type, negativity of CD34 and CD56 expression and, probably, CBF AML. Taken together, these findings can be useful in the management of AML patients. Novel therapeutic approaches could be designed in order to manipulate the immune microenvironment, reversing the “do not eat me signal” of CD200. Anti-CD200 antibodies have demonstrated *in vitro* efficacy [49] and are under investigation in chronic lymphocytic leukemia [50]. Moreover many drugs able to deactivate MDSC [51, 52], to block their development [53, 54] or to deplete them [54, 55] have been developed in the last years and will probably deeply change the therapeutic scenarios of hematologic malignancies.
